# Supplementary material for: Protein control of photochemistry and transient intermediates in phytochromes
Source: Nat Commun. 2022 Nov 11;13:6838. doi: 10.1038/s41467-022-34640-8 (PMC9652276; doi:10.1038/s41467-022-34640-8)
Supplement: Supplementary file 1 — Supplementary Information [file 41467_2022_34640_MOESM1_ESM.pdf]

# Supplementary Information: Protein control of photochemistry and transient intermediates in phytochromes

Giacomo Salvadori[1*], Veronica Macaluso[1], Giulia Pellicci[1], Lorenzo Cupellini[1], Giovanni Granucci[1], and Benedetta Mennucci[1*]

[1]*Department of Chemistry and Industrial Chemistry, University of Pisa, Via G. Moruzzi 13, 56126 Pisa, Italy*
[*]*email: giacomo.salvadori@phd.unipi.it; benedetta.mennucci@unipi.it*

## Supplementary Figures

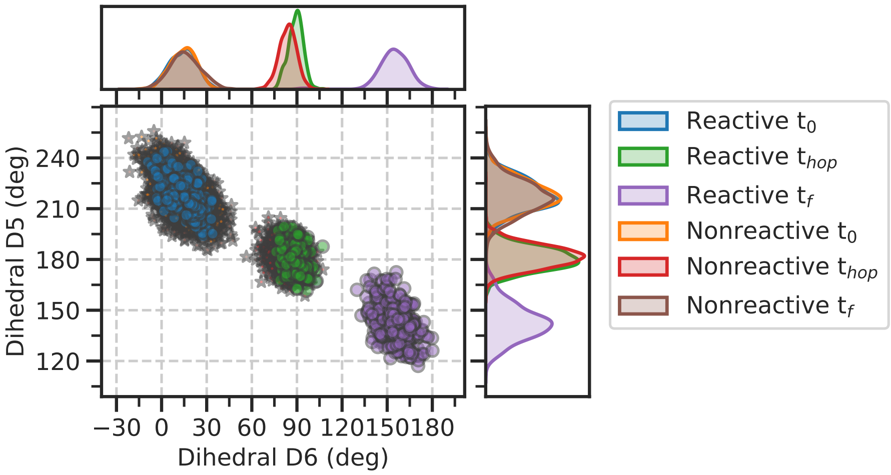


Supplementary Figure 1: Scatter plot of the dihedral angles D5 and D6 (for both reactive and non-reactive trajectories) at the starting conditions (orange and blue), at the $S_1 \rightarrow S_0$ hop (green and red) and at the end of the simulation (violet and brown). It has to be noted that the "non reactive $t_f$" trajectories have the same distribution as the "non reactive $t_0$" ones.

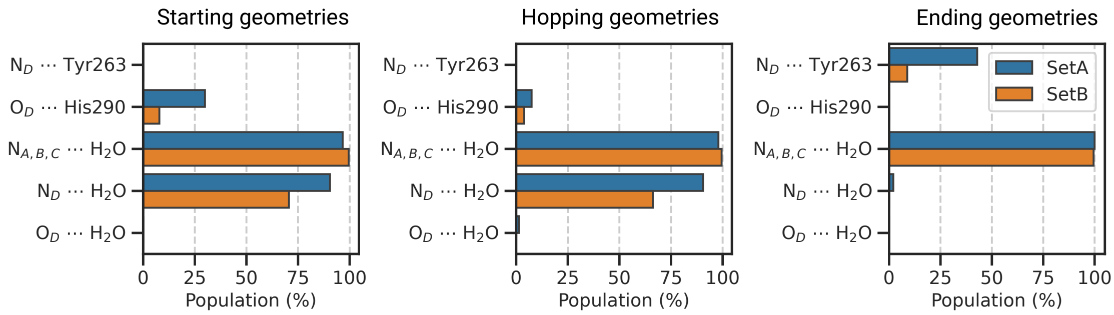


Supplementary Figure 2: Probabilities for H-bonds involving the chromophore at the starting conditions (starting structures), at the $S_1 \rightarrow S_0$ hop (Hopping structures), and at the end of the simulation (Final structures). This analysis was performed on the reactive trajectories.

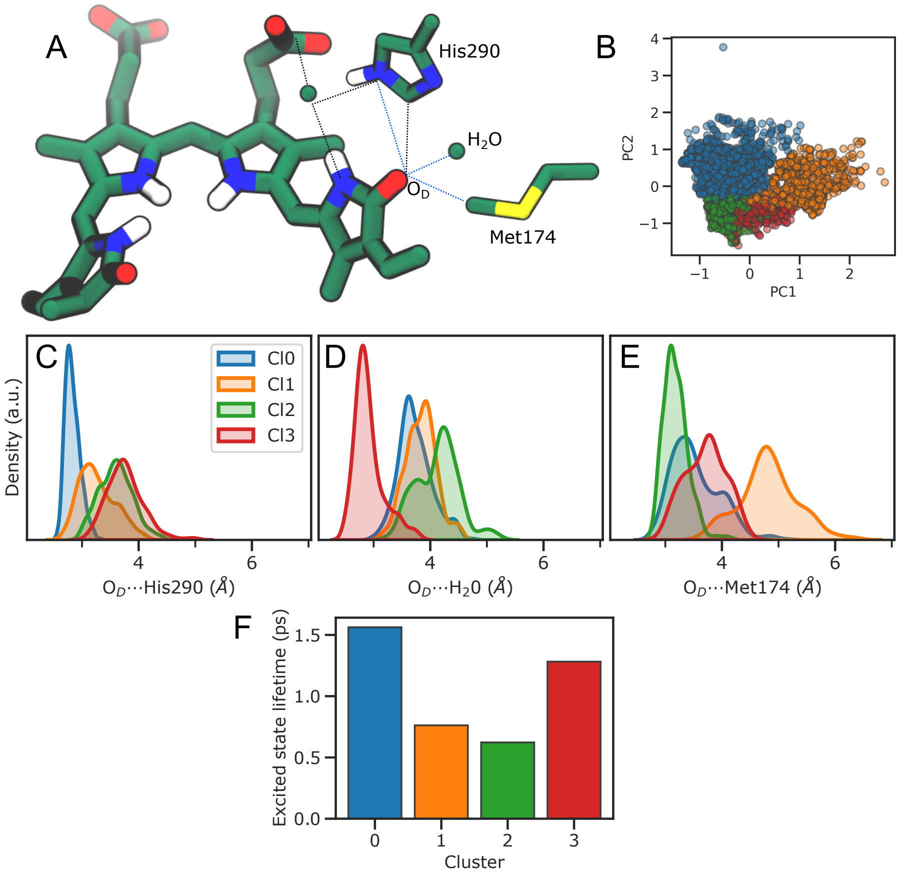


Supplementary Figure 3: Analysis of the SH initial conditions and relation to the lifetime. (A) Depiction of the distances used in the classification of the SH initial conditions. (B) Clustering visualized in PCA space. Points differently colored correspond to different clusters. (C)-(E) Distribution of the distances that separate the four clusters (blue lines in panel A). (F) Excited state lifetimes for each cluster

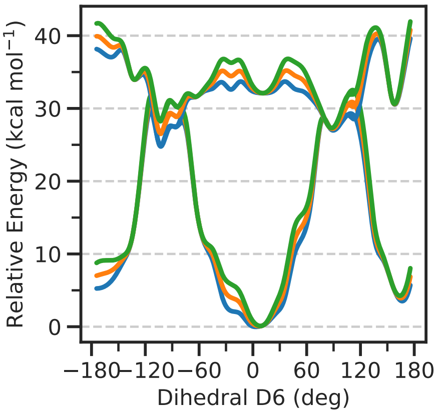


Supplementary Figure 4: Potential energy curves ($S_0$ and $S_1$) along the dihedral D6. Geometries were obtained by optimization of all coordinates, but D6, at $S_1$ electronic state. On each curve, we added the following function: $k \cdot (1 - \cos(5 \cdot (x - 10.6))$, where $k$ is equal to 0 eV (blue), 0.04 eV (yellow), 0.08 eV (green), and 0.10 eV (violet).

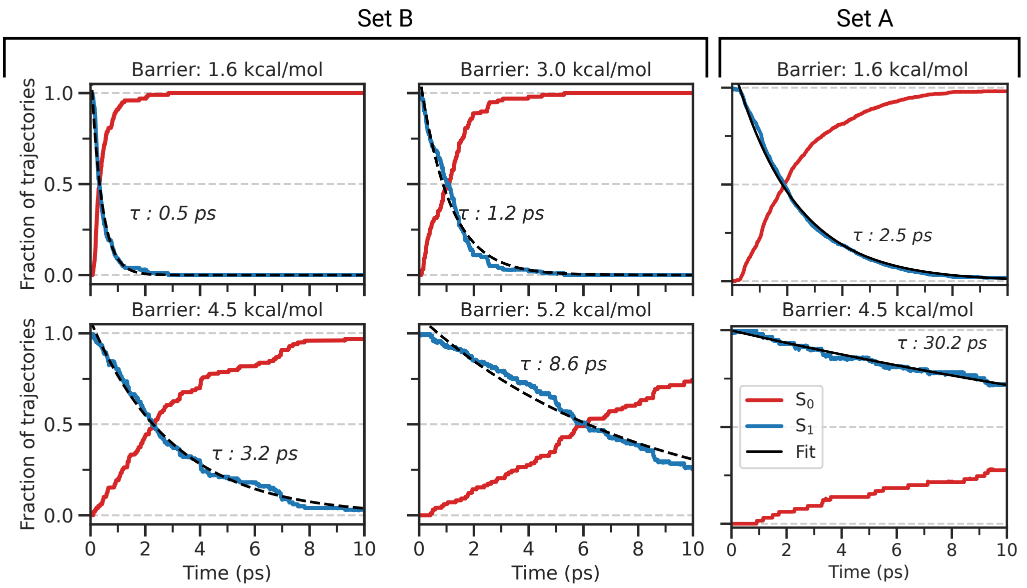


Supplementary Figure 5: Time evolution of the electronic state populations, together with the fit of the $S_1$ population according to an exponential function, for both Set A and Set B. We represented both the "original" evolution (see "Barrier: 1.6 kcal mol$^{-1}$" cases and Supplementary Table 1) and the population evolutions with the additional potentials on dihedral D6 (see "Barrier: 3.0/4.5/5.2 kcal mol$^{-1}$" cases for Set B, and "Barrier: 4.5 kcal mol$^{-1}$" case for Set A. The excited-state lifetimes were computed by fitting the adiabatic state populations through a simple kinetic model reported in Supplementary Methods 3. For Set B, we have used 99,99, and 98 trajectories to generate the lifetimes when the barrier is 3.0, 4.5, and 5.2 kcal, respectively. For Set A, we have used 68 trajectories to generate the lifetime when the barrier is 4.5 kcal mol$^{-1}$. The number of trajectories used when the barrier is 1.6 kcal mol$^{-1}$ are reported in Supplementary Table 1.

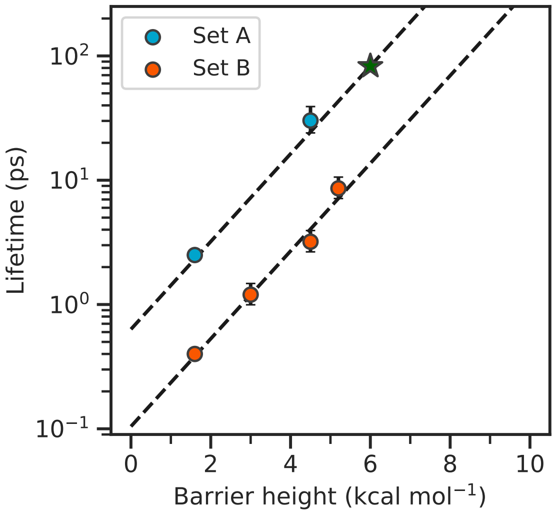


Supplementary Figure 6: Excited-state lifetimes as a function of the barrier magnitudes for both Set A and Set B together with their 95 % confidence intervals. We fitted the four cases of Set B with a line of slope $m$. The same slope $m$ is used to connect the two points of set A. The green star was obtained by extrapolating the data in Set A. The population fittings shown in Supplementary Fig. 5 give us the excited-state lifetimes, which were used as centres for the error bars. For Set B, we have used 99,99, and 98 trajectories to generate the lifetimes when the barrier is 3.0, 4.5, and 5.2 kcal, respectively. For Set A, we have used 68 trajectories to generate the lifetime when the barrier is 4.5 kcal mol$^{-1}$. The number of trajectories analysed when the barrier is 1.6 kcal mol$^{-1}$ are reported in Supplementary Table 1.

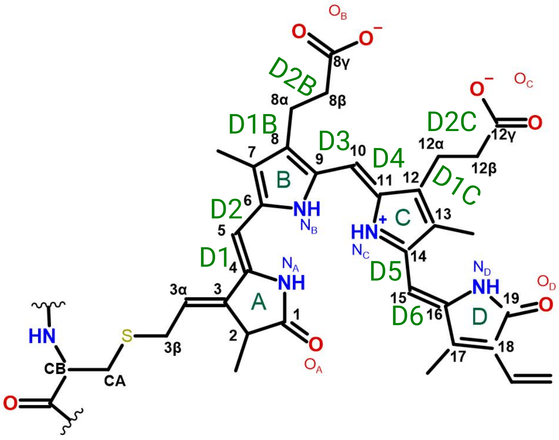


| Feature | Atoms sequence |
|---|---|
| D6 | $C_{14} - C_{15} = C_{16} - N_D$ |
| D5 | $N_C = C_{14} - C_{15} = C_{16}$ |
| D4 | $N_C - C_{11} = C_{10} - C_9$ |
| D3 | $N_B - C_9 - C_{10} = C_{11}$ |
| D2 | $N_B - C_6 - C_5 = C_4$ |
| D1 | $N_A - C_4 = C_5 - C_6$ |
| D1B | $C_7 - C_8 - C_{8\alpha} - C_{8\beta}$ |
| D2B | $C_8 - C_{8\alpha} - C_{8\beta} - C_{8\gamma}$ |
| D1C | $C_{11} - C_{12} - C_{12\alpha} - C_{12\beta}$ |
| D2C | $C_{12} - C_{12\alpha} - C_{12\beta} - C_{12\gamma}$ |
| Angle C/D rings | $C_{14} - C_{15} = C_{16}$ |

Supplementary Figure 7: Biliverdin chemical structure and definition of the main dihedral angles.

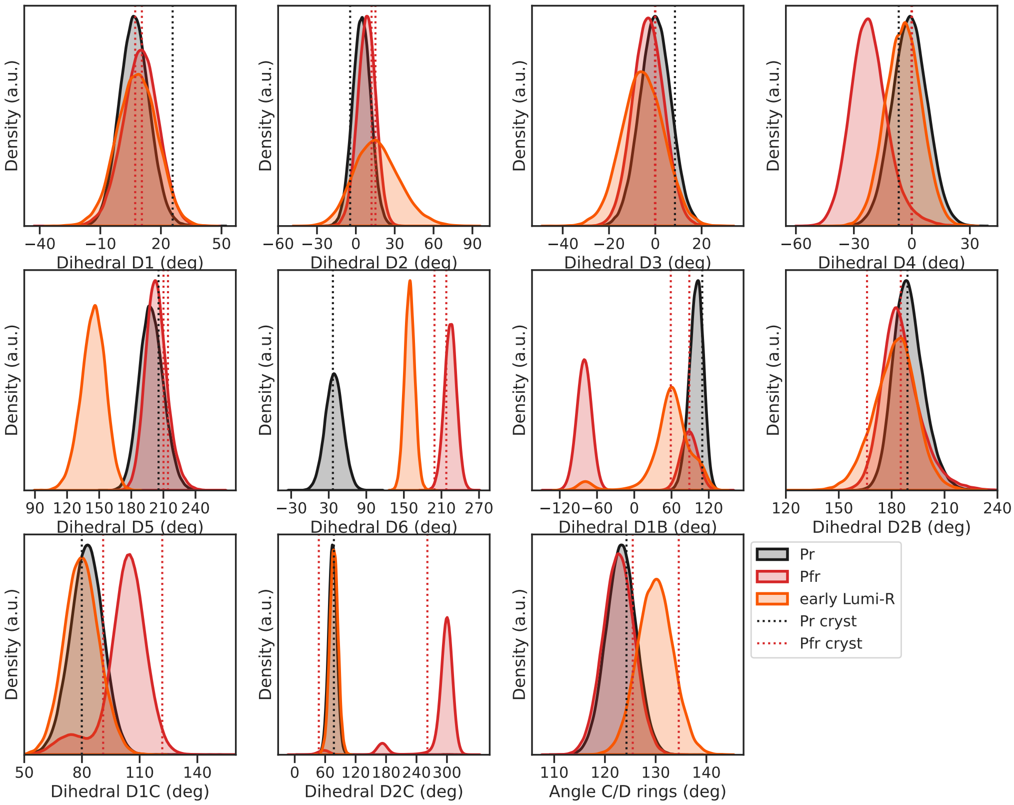


Supplementary Figure 8: Distribution of the structural features for the QM/MM MDs. For comparison, we reported the respective values for the Pr and Pfr states (both crystal-structure and MD-equilibrium values[1,2]).

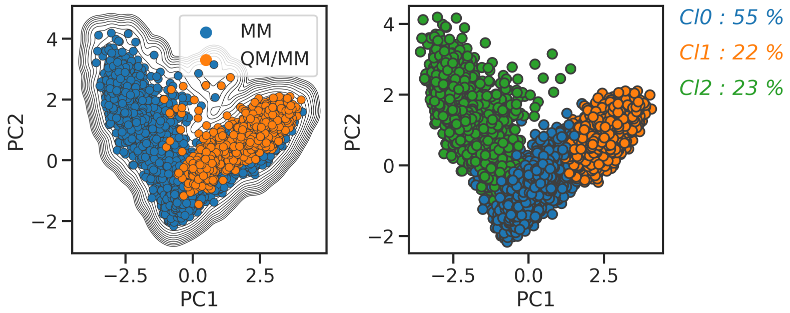


Supplementary Figure 9: Left panel: PCA analysis performed on both the MM (blue) and QM/MM (orange) MD simulations. Right panel: Clustering of the MM and QM/MM MDs simulations, visualized in PCA space. Points differently colored correspond to different clusters: cluster 0 (Cl0) in blue, cluster 1 (Cl1) in orange, and cluster 2 (Cl2) in green).

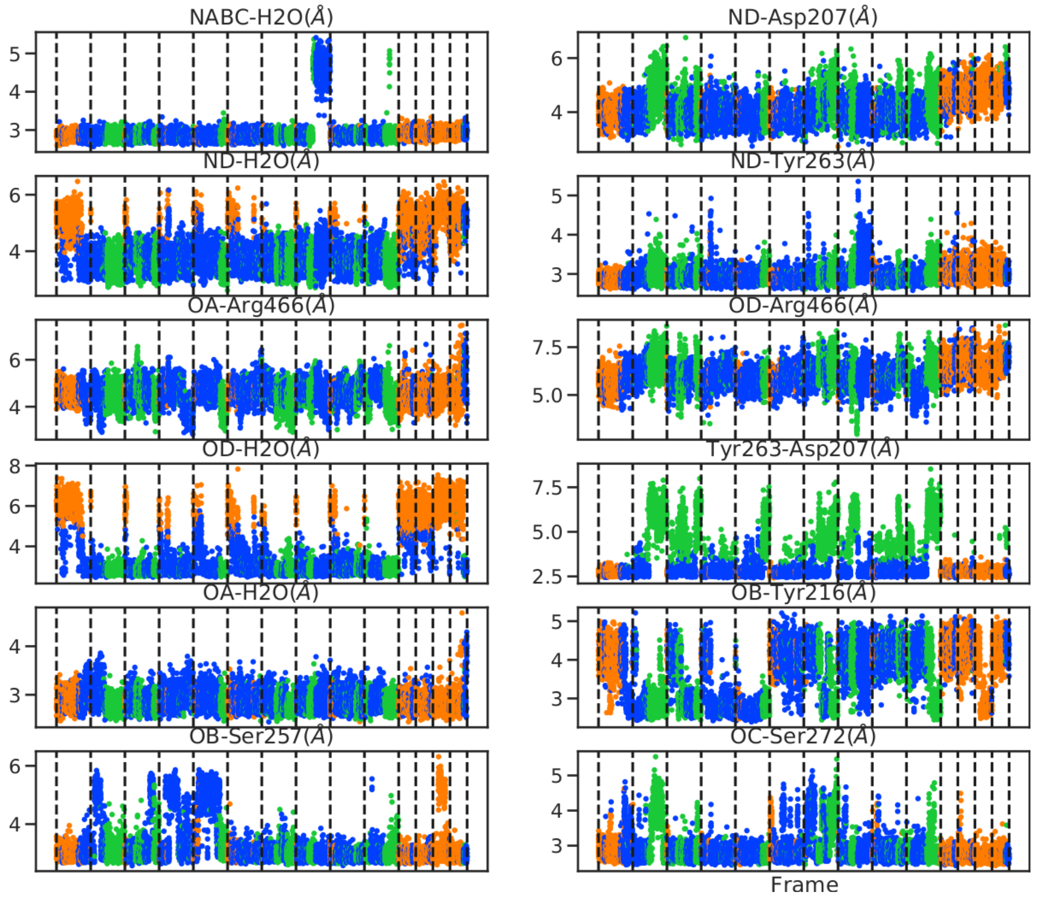


Supplementary Figure 10: Trend of the intermolecular distances involving key-residues in the chromophore binding pocket for the QM/MM MDs and the first 10 ns of the MM MDs. Each color represents a different cluster. Black dashed lines separate the different MD replicas. The first 10 MDs are MM MDs, while the last 4 MDs are QM/MM MDs.

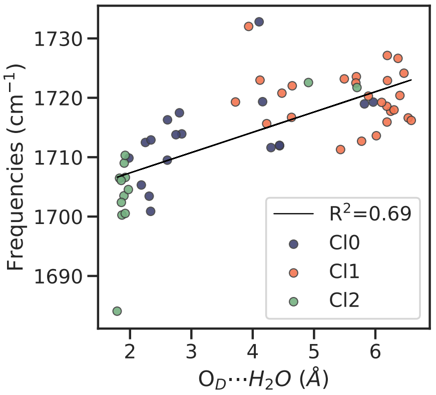


Supplementary Figure 11: Scatter plot for the $CO_D$ stretching frequency and the hydrogen bonding distance between oxygen ($O_D$) and water.

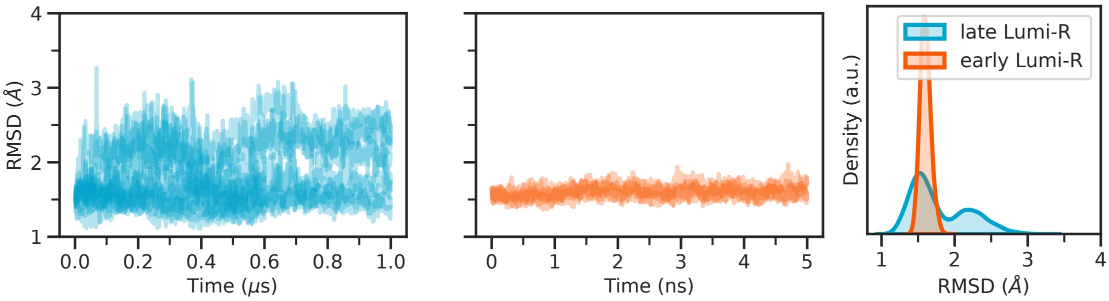


Supplementary Figure 12: Root-mean-square displacement (RMSD) of the chromophore binding pocket (and distributions) using as reference the Pr crystal structure. In blue the late Lumi-R, characterized by MM MDs (1 $\mu$s-long each), in orange the early Lumi-R, characterized by QM/MM MDs (5 ns-long each).

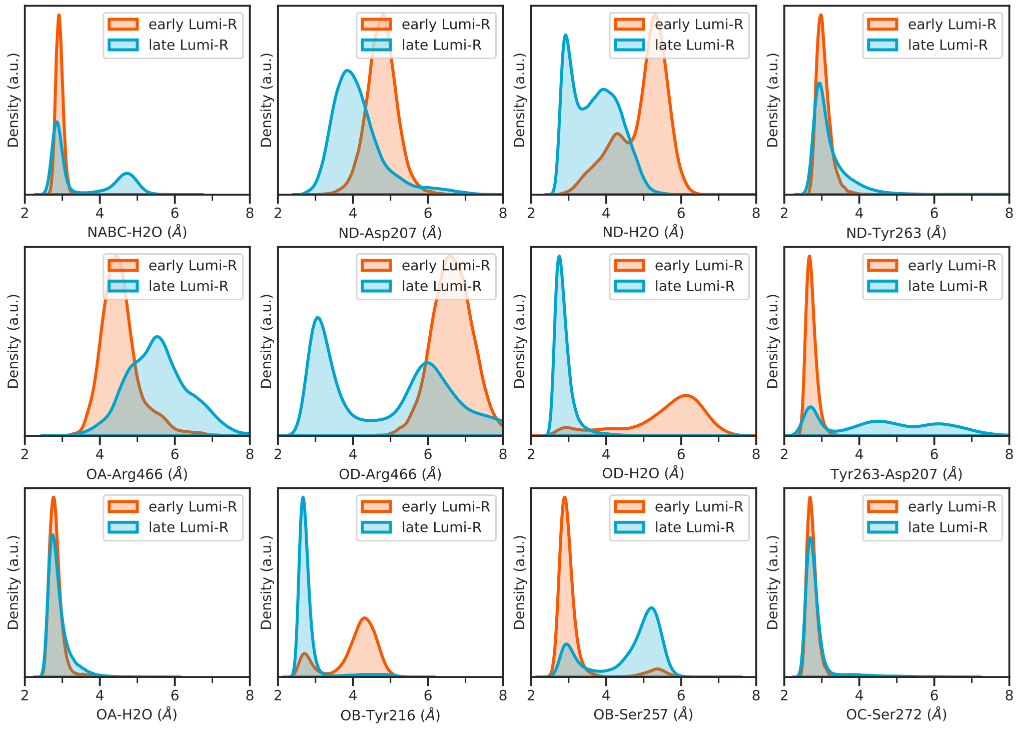


Supplementary Figure 13: Distribution of the minimum intermolecular distances involving key-residues in the chromophore binding pocket for the early (orange) and late (blue) Lumi-R.

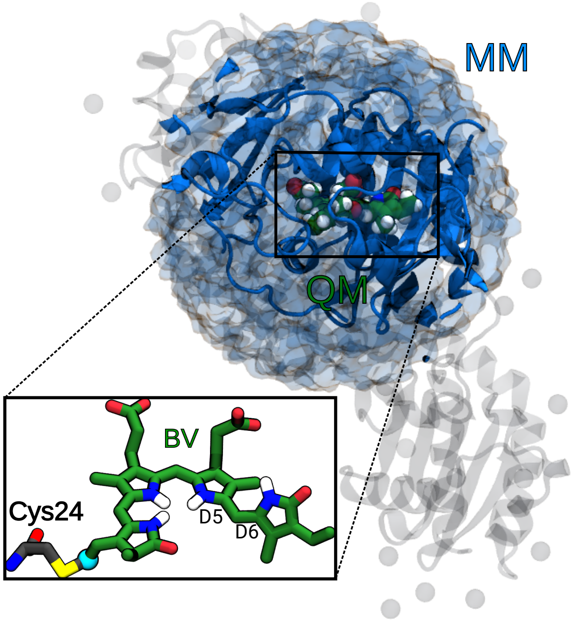


Supplementary Figure 14: Depiction of the model system considered in QM/MM surface hopping simulations and chemical structure of the chromophore with indication of the connection atom in cyan ($C_{3\beta}$ carbon atom in Fig.7) and definition of the D5 and D6 dihedral angles involved in the photoisomerization

## Supplementary Table

Supplementary Table 1: $\alpha$, and $\beta$ refer to two different simulations computed forbidding hops when the energy gap between electronic states $S_0$ and $S_1$ is larger than 1.5 eV, and 0.5 eV, respectively. Number of trajectories, quantum yield, and lifetimes, $\tau$ (ps). "Long-lived trajs" represents the number of trajectories with an excited-state lifetime greater than 10 ps. The lifetime, $\tau$, was obtained by fitting the decay of the $S_1$ electronic state with an exponential function (Supplementary Methods 3). Time at the $S_1$-to-$S_0$ hop, dihedral angles D5 and D6 (degree), and $S_0$-$S_1$ energy gap $\Delta$E (kcal mol$^{-1}$) averaged over $S_1 \rightarrow S_0$ hop events. Each average was computed separately for reactive and nonreactive trajectories.

| | | Set A | | Set B | |
|---|---|---|---|---|---|
| | | $\alpha$ | $\beta$ | $\alpha$ | $\beta$ |
| | $N_{trajs}$ | 997 | 968 | 1445 | 1444 |
| | Long-lived trajs | 4 | 18 | 0 | 0 |
| | $\tau$ | 2.24 | 2.54 | 0.48 | 0.48 |
| | Quantum Yield | 0.111±0.001 | 0.160±0.001 | 0.133±0.001 | 0.141±0.001 |
| Reactive | $\text{Time}_{S_1 \rightarrow S_0}$ | 2470±364 | 2660±357 | 470±61 | 467±58 |
| | $\langle \text{D6} \rangle_{S_1 \rightarrow S_0}$ | 89±1 | 88±1 | 90±1 | 90±1 |
| | $\langle \text{D5} \rangle_{S_1 \rightarrow S_0}$ | 178±1 | 179±1 | 182±1 | 182±1 |
| | $\langle \Delta \text{E} \rangle_{S_1 \rightarrow S_0}$ | 1.3±0.2 | 1.3±0.2 | 1.3±0.2 | 1.3±0.2 |
| Non Reactive | $\text{Time}_{S_1 \rightarrow S_0}$ | 2150±112 | 2397±134 | 496±24 | 503±25 |
| | $\langle \text{D6} \rangle_{S_1 \rightarrow S_0}$ | 74±1 | 83±1 | 83±1 | 84±1 |
| | $\langle \text{D5} \rangle_{S_1 \rightarrow S_0}$ | 183±1 | 180±1 | 183±1 | 183±1 |
| | $\langle \Delta \text{E} \rangle_{S_1 \rightarrow S_0}$ | 10.2±0.6 | 4.5±0.2 | 4.7±0.3 | 3.7±0.2 |

## Supplementary Note 1: Analysis of the role of H-bond interactions in the excited state lifetimes

To get insight into the role of the protein pocket in determining the excited state lifetimes, we ran 881 additional SH trajectories using for their initial conditions (nuclear coordinates and momenta) the ground state QM/MM trajectories described in the Methods section of the main text. These trajectories were pooled with sets A and B and categorized based on the distances between the D-ring and the closest residues at the initial conditions (Supplementary Fig. 3A). Due to the stochastic nature of Surface Hopping, we cannot analyze individual SH trajectories. Instead, we pool together the trajectories with similar initial configurations, and we compute the excited-state lifetime by fitting the average populations of each trajectory ensemble.

A hierarchical agglomerative algorithm was used together with principal component analysis (PCA) to categorize initial conditions into 4 clusters (represented in Supplementary Fig. 3B on the first two principal components). Each cluster is characterized by a different set of distances (Supplementary Fig. 3C-E). In particular, cluster 0 shows a small distance between the D-ring carbonyl ($O_D$) and His290, whereas cluster 3 is characterized by a small distance between $O_D$ and a water molecule. Instead, clusters 1 and 2 show larger values for both distances. Therefore, only clusters 0 and 3 present a hydrogen bond to $O_D$. Analyzing the lifetimes (Supplementary Fig. 3F), it is clear that both clusters 0 and 3 have a significantly longer excited-state lifetime than clusters 1 and 2. Visual inspection of the structures in cluster 3 revealed that the water molecule makes a H-bond interaction with both $O_D$ and His290. Therefore, the $O_D \cdots$ His290 H-bond can be either direct (cluster 0) or water-mediated (cluster 3). In either case, the H-bond results in a substantially longer excited-state lifetime.

## Supplementary Note 2: Analysis of the early Lumi-R intermediate

As described in the main text, in order to validate our putative early Lumi-R intermediate, we have computed the IR spectrum starting from configurations sampled by the QM/MM MDs. To achieve a detailed overview of the configurational space, we relied on the first time window (10 ns) sampled by MM MDs. To have a more quantitative comparison of the configurational space spanned by QM/MM and MM MDs, we have used a principal component analysis (PCA) based on intermolecular distances involving key-residues in the chromophore binding pocket (Supplementary Fig. 15).

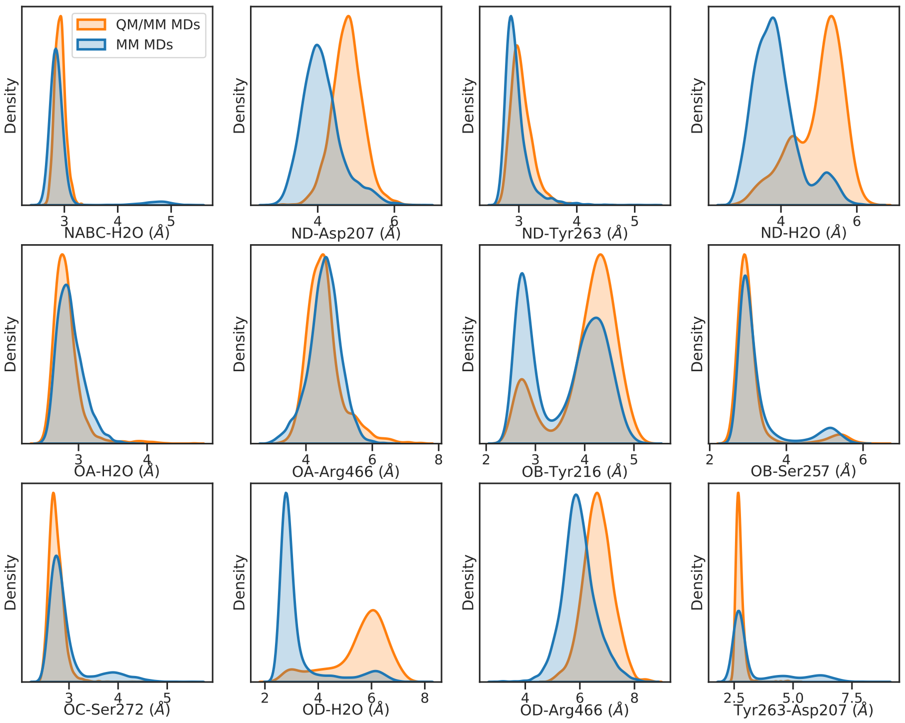


Supplementary Figure 15: Distribution of the minimum intermolecular distances involving key-residues in the chromophore binding pocket for the QM/MM MDs (orange) and the first 10 ns of the MM (blue) MDs.

We used 10000 structures extracted from the MM MDs and 2000 structures from QM/MM MDs. This analysis reveals that the QM/MM MDs explore a subset of the MM MD configurations (Supplementary Fig. 9).

Then, we have used a hierarchical agglomerative algorithm on all 12 principal components (Supplementary Fig. 16A) employing Ward's method for performing each merge (Supplementary Fig. 16B). The number of clusters were chosen monitoring the Calinsky-Harabasz, the mean silhouette, and the Davies Bouldin scores (Supplementary Fig. 16C). Thus, a final number of three clusters was chosen and projected in the PCA space (Supplementary Fig. 9).

PCA and clustering analysis were performed with Scikit-Learn [3].

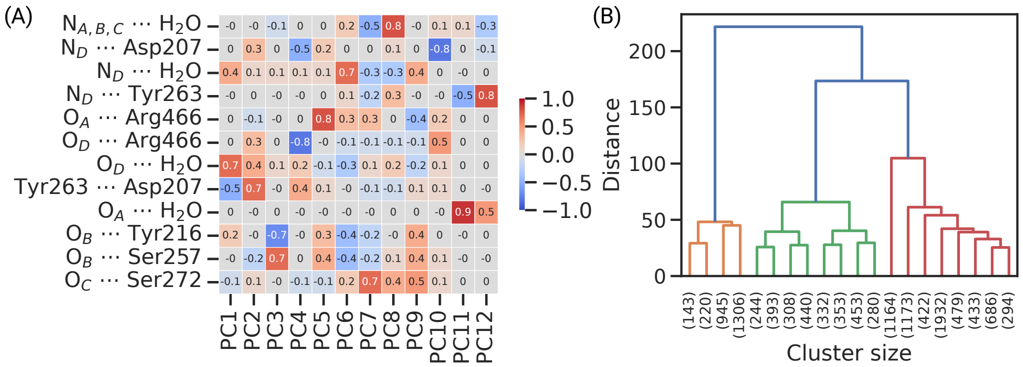


(C)

| N Cluster | Silhouette score | Calinski Harabasz score | Davies Bouldin score |
|---|---|---|---|
| 3 | 0.35 | 6633.19 | 1.15 |
| 4 | 0.33 | 5946.58 | 1.06 |
| 5 | 0.30 | 5032.42 | 1.44 |
| 6 | 0.24 | 4483.65 | 1.51 |

Supplementary Figure 16: (A) Composition of the twelve principal components according to the distances used for the PCA analysis. (B) Hierarchical agglomerative algorithm (C) Scores monitored to select the optimal number of clusters.

# Supplementary Methods 1: Selection of the semiempirical QM method

The simulation of the photochemical process was carried out by means of the mixed quantum-classical surface hopping (SH) method, pioneered by Tully[4]. In particular, we employed a hybrid QM/MM scheme with electrostatic embedding. As it regards the QM part, we relied on semiempirical (SE) Hamiltonians. In the following we report on the selection of the SE method and its benchmark validation.

As a preliminary static analysis we have investigated the ground and excited states minima. In order to reduce the computational effort, a simplified chromophore was selected as model system: the thioether, the methyl group on the ring A, and the propionyl groups were replaced by hydrogen atoms (Supplementary Fig. 17).

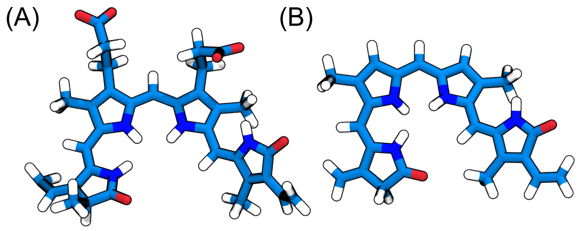


Supplementary Figure 17: (A) Biliverdin IX$\alpha$ (B) Model system used to select the computational method

(TD)DFT optimizations were performed at B3LYP[5] and CAM-B3LYP[6] level of theory, respectively, on such model system. In both cases the 6-31G(d) basis set was used. The optimization of the excited state shows a local minimum close to the ground-state equilibrium geometry, i.e. at a D6 dihedral angle of about 7 degrees (Supplementary Table. 2). Ground and excited state optimizations were also repeated in protein using a QM/MM approach treating His290, His260, BV, Cys24, and two water molecules at QM level. For the ground state optimizations the QM subsystem was described at B3LYP-D3/6-31G(d) level of theory[7], while the excited state ones were performed at TDCAM-B3LYP/6-31+G(d) level of theory. In both cases, the protein was kept frozen and described by the ff14SB force field[8]. For the excited state we selected the CAM-B3LYP functional because, in a previous work,[1] it showed to be able to correctly reproduce the absorption and circular dichroism spectra of the Pr state. For these calculations, we used the ONIOM(QM:MM) scheme[9], implemented in the Gaussian 16 suite of programs[10].

The QM/MM results are in agreement with the ones obtained in vacuum, i.e. the excited state shows a minimum energy point close to the Franck Condon region (Supplementary Table. 2), which is in line with the literature[11,12]. To get more insight into the description of the excited state, the Natural Transition Orbital (NTO) analysis[13] was performed. This analysis shows that the first excited state is characterized by a $\pi \rightarrow \pi^*$ "short-range" excitation, localized on the chromophore (Supplementary Fig. 18).

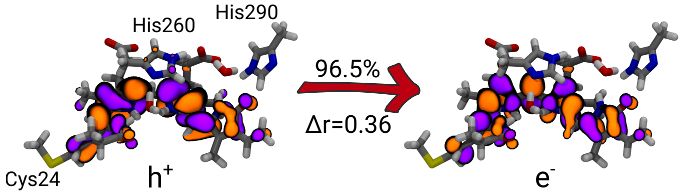


Supplementary Figure 18: CAM-B3LYP NTO orbital pairs, "hole" (h) and "electron" ($e^-$), computed for Pr crystal structure for the $S_0 \rightarrow S_1$ transition, together with the hole-electron distance ($\Delta$r)[14]

Then, we tested a series of semiempirical Hamiltonians (AM1, PM3, PM6, PM7, RM1, MNDO, OM2 and OM3) in combination with a CAS-CI description, by performing ground and excited states geometry optimizations of the model system in vacuum. With the exception of OM2 and OM3, whose calculations were made with MNDO [15], MOPAC2002 [16] was used. As it regards AM1, PM6, PM3, RM1 and MNDO, active spaces (4,5), (6,5), and (6,6) were used, while for OM2 and OM3 we used a much larger active space, i.e. (8,8), (12,10) and (16,12). All SE methods which showed free torsion around the dihedrals D3 and/or D6 in the excited state were discarded. From this initial pool of candidates, only the AM1 Hamiltonian in combination the multi-reference floating occupation molecular orbital-complete active space configuration interaction (FOMO-CASCI) scheme[17,18] and a (6,6) active space was retained.

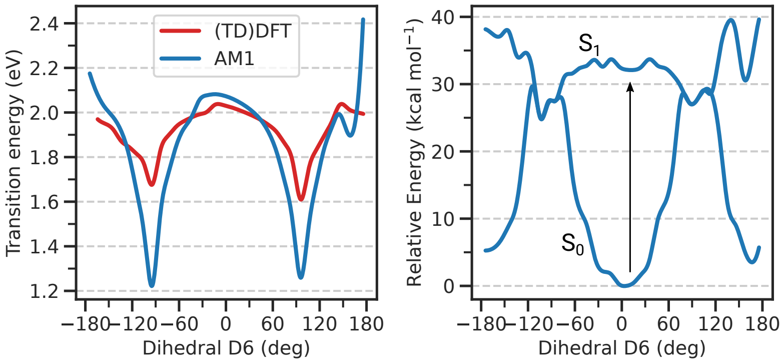


Supplementary Figure 19: Left panel: Transition energy $S_0 \rightarrow S_1$ computed on the ground state-optimized geometries. We reported both the semiempirical (blue) and the (TD)DFT (red) trends. The latter only in the Frank Condon region. Right Panel: PES resulting from an optimization of the excited state $S_1$ at AM1 / FOMO-CASCI / 6,6 level of theory.

The ground-state geometry of the model system obtained with B3LYP level of theory is well reproduced at AM1 level (Supplementary Table 2). In Supplementary Fig. 19 we show the $S_0$-$S_1$ transition energy evaluated as a function of the D6 dihedral at the ground state optimized geometries. We notice the good agreement between the TDCAM-B3LYP results and the semiempirical ones. We also report the $S_0$ and $S_1$ energies obtained at AM1 level optimizing the $S_1$ state. The resulting AM1-PES (Supplementary Fig. 19) shows that the excited state presents a local minimum in proximity of the Franck Condon region (Supplementary Table. 2) and, more importantly, a conical intersection between $S_0$ and $S_1$ at about 90 degrees of torsion.

## Supplementary Methods 2: Surface Hopping trajectories

The electronic energies, wavefunctions and non-adiabatic couplings were evaluated "on the fly" during the integration of the nuclear trajectories, with the AM1 Hamiltonian in combination with the FOMO-CI scheme[18,19]. For all non-adiabatic trajectories an energy conservation threshold of c.a. 5 kcal mol$^{-1}$ was set. To reduce the computational cost, trajectories were stopped when one of the following two criteria was fulfilled: (i) the trajectory has been running on the ground state surface for 500 fs; (ii) the total simulation time is larger than 10 ps. After a backward hop, we rescale the component of the velocity vector parallel to the non-adiabatic coupling vector between the old and the new active states. Regarding forward hops, the nuclear velocity vector is rescaled by keeping its direction unaltered. The reason for this difference is twofold. First, the QM system has always enough kinetic energy to compensate for backward hops, once on the ground state: it is, therefore, essential to consider that only the velocity vector component parallels to the non-adiabatic couplings should be rescaled. Second, we judged the computation of non-adiabatic

Supplementary Table 2: Structural analysis carried out on the BV model system in protein and in vacuum, following geometry optimization of the ground and excited states. For the nomenclature of the atoms, see Supplementary Fig. 7.

| | Vacuum AM1 | | Vacuum DFT | | Protein DFT | |
|---|---|---|---|---|---|---|
| | $S_0$ | $S_1$ | $S_0$ | $S_1$ | $S_0$ | $S_1$ |
| $C_{14} - C_{15} = C_{16} - N_D$ (D6) | 5.6 | 11.3 | 5.8 | 7.5 | 17.6 | 20.4 |
| $N_C - C_{14} - C_{15} = C_{16}$ (D5) | -144.1 | -159.6 | -155.6 | -156.2 | -154.0 | -157.3 |
| $N_C - C_{11} - C_{10} = C_9$ (D4) | -8.7 | -29.3 | -10.7 | -14.2 | -1.1 | 1.4 |
| $N_B - C_9 = C_{10} - C_{11}$ (D3) | -9.3 | 0.4 | -9.5 | -8.4 | -0.47 | -3.6 |
| $N_B - C_6 - C_5 = C_4$ (D2) | 21.8 | 4.5 | 15.4 | 13.2 | 13.7 | 12.7 |
| $N_A - C_4 = C_5 - C_6$ (D1) | 4.3 | 10.2 | 6.8 | 9.4 | 11.3 | 12.9 |

couplings not worth the computational effort for forwarding hops (i.e., $S_1$-to-$S_0$), which are always allowed (notice that the time evolution of the electronic wave function is performed through the evaluation of wave function overlaps, so the non-adiabatic couplings have to be evaluated primarily for hops).

In all simulations, evaporation of water molecules from the surface of the water sphere is precluded by adding, for each atom, a confining boundary potential:

$$V_{\mathrm{conf}}(R) = \begin{cases} 0 & \text{if } R \le R_{wall} \\ \frac{1}{2}(R - R_{wall})^2 & \text{if } R > R_{wall} \end{cases}$$

where K = 0.544 eV Å$^{-2}$, R is the distance of the atom from the center of the sphere and $R_{wall} =$ 26Å.

# Supplementary Methods 3: Kinetic model

We have fitted the time evolution of the $S_1$ electronic excited state population with a delayed exponential function:

$$P_{S_1}(t) = \begin{cases} \exp\left[-\frac{t-t_0}{\tau'}\right] & \text{for } t > t_0 \\ 1.0 & \text{for } t < t_0 \end{cases}$$

where $t_0$ is a delay time.

The overall lifetime of $S_1$ (reported in Supplementary Table 3 and Supplementary Table 1) was obtained as $\tau = \tau' + t_0$.

Supplementary Table 3: Definition of the $S_1$ excited state lifetimes (ps). $t_0$ represents the delay time. The overall lifetime was obtained as $\tau = \tau' + t_0$.

| | Set A | Set B |
|---|---|---|
| $t_0$ | 0.23 | 0.07 |
| $\tau'$ | 2.01 | 0.41 |
| $\tau$ | 2.24 | 0.48 |

## Supplementary Methods 4: MD simulations of the intermediates

Configurations of the photoproduct obtained from the last frame of the reactive SH trajectories were used to propagate adiabatic MD simulations of the system in the ground state in search of the Lumi-R intermediates. In these simulations, we reintroduced a full solvation of the protein using a truncated octahedron water box. Both QM/MM and MM MD simulations were performed.

For the MM simulations, the solvated system was subjected to 400 steps of energy minimization using steepest descent and other 1600 steps using conjugate gradient. Then, a gradually heating to 300 K in a NVT ensemble in 400 ps followed, restraining the movement of the chromophore and the protein backbone with a 4 kcal $mol^{-1}Å^{-2}$ harmonic potential. The same constraints were maintained for the following 1 ns-long equilibration step. The production run was carried out without any restraint for 1 $\mu$s in the NPT ensemble.

For the QM/MM simulations, the solvatated system was subjected to 400 steps of energy minimization using steepest descent and other 600 steps using conjugate gradient. Then, a gradually heating to 300 K in a NVT ensemble in 20 ps followed, restraining the movement of the chromophore and the protein backbone with a 4 kcal $mol^{-1}Å^{-2}$ harmonic potential. It followed a 30 ps NPT equilibration run with the same restraints on the chromophore and all the protein backbone. The production run was run without any restraint in the NPT ensemble for 5ns.

All simulations were performed applying the particle mesh Ewald (PME) truncation method (with a short-range cut-off of 10Å), an integration step of 2 fs, the SHAKE algorithm, a Langevin thermostat with a friction coefficient of 1 $ps^{-1}$, and the Monte Carlo barostat for NPT simulations. All molecular dynamics simulations were performed with AMBER18[20].

## Supplementary Methods 5: Calculation of the IR spectra

For the calculation of the IR spectra of the intermediate, a three-step protocol was used:

1. Full MM optimization of the chromophore and the residues within 4 Å around it. The residues outside this shell were kept frozen;
2. QM/MM optimization where the environment treated at MM level was kept frozen, while the QM part was described at B3LYP+D3/6-31G(d) level of theory;
3. A refinement of the previous QM/MM optimization using the B3LYP+D3/6-311G(d,p) level of theory for the QM part.

The frequencies obtained from this last optimization were used to compute the IR spectra. A lorentzian function with a HWHM of 4 $cm^{-1}$ was used as lineshape to obtain the spectrum of each frame. The final spectrum was scaled by a factor of 0.96 to account for anharmonic effects.

## Supplementary References


[1] Macaluso, V., Cupellini, L., Salvadori, G., Lipparini, F. & Mennucci, B. Elucidating the role of structural fluctuations, and intermolecular and vibronic interactions in the spectroscopic response of a bacteriophytochrome. *Phys. Chem. Chem. Phys.* **22**, 8585–8594 (2020).

[2] Macaluso, V., Salvadori, G., Cupellini, L. & Mennucci, B. The structural changes in the signaling mechanism of bacteriophytochromes in solution revealed by a multiscale computational investigation. *Chem. Sci.* **12**, 5555–5565 (2021).

[3] Pedregosa, F. *et al.* Scikit-learn: Machine learning in python. *J. Mach. Learn. Res.* **12**, 2825–2830 (2011).

[4] Tully, J. C. Molecular dynamics with electronic transitions. *J. Chem. Phys.* **93**, 1061–1071 (1990).

[5] Becke, A. D. Density-functional thermochemistry. III. The role of exact exchange. *J. Chem. Phys.* **98**, 5648–5652 (1993).

[6] Yanai, T., Tew, D. P. & Handy, N. C. A new hybrid exchange–correlation functional using the Coulomb-attenuating method (CAM-B3LYP). *Chem. Phys. Lett.* **393**, 51–57 (2004).

[7] Grimme, S., Antony, J., Ehrlich, S. & Krieg, H. A consistent and accurate ab initio parametrization of density functional dispersion correction (DFT-d) for the 94 elements h-pu. *J. Chem. Phys.* **132**, 154104 (2010).

[8] Maier, J. A. *et al.* ff14SB: Improving the Accuracy of Protein Side Chain and Backbone Parameters from ff99SB. *J. Chem. Theory Comput.* **11**, 3696–3713 (2015).

[9] Chung, L. W. *et al.* The ONIOM Method and Its Applications. *Chem. Rev.* **115**, 5678–5796 (2015).

[10] Frisch, M. J. *et al.* Gaussian 16 Revision A.03 (2016). Gaussian Inc. Wallingford CT.

[11] Slavov, C. *et al.* The interplay between chromophore and protein determines the extended excited state dynamics in a single-domain phytochrome. *Proc. Natl. Acad. Sci. USA* **117**, 16356–16362 (2020).

[12] Altoè, P. *et al.* Deciphering intrinsic deactivation/isomerization routes in a phytochrome chromophore model. *J. Phys. Chem. B* **113**, 15067–15073 (2009).

[13] Martin, R. L. Natural transition orbitals. *J. Chem. Phys.* **118**, 4775–4777 (2003).

[14] Guido, C. A., Cortona, P. & Adamo, C. Effective electron displacements: A tool for time-dependent density functional theory computational spectroscopy. *J. Chem. Phys.* **140**, 104101 (2014).

[15] Thiel, W. Program mndo2004 (2004). Muelheim.

[16] Stewart, J. J. Mopac2002 (2002). Fujitsu Limited: Tokyo, Japan.

[17] Granucci, G., Persico, M. & Toniolo, A. Direct semiclassical simulation of photochemical processes with semiempirical wave functions. *J. Chem. Phys.* **114**, 10608–10615 (2001).

[18] Persico, M. & Granucci, G. An overview of nonadiabatic dynamics simulations methods, with focus on the direct approach versus the fitting of potential energy surfaces. *Theor. Chem. Acc.* (2014).

[19] Granucci, G. & Toniolo, A. Molecular gradients for semiempirical ci wavefunctions with floating occupation molecular orbitals. *Chem. Phys. Lett.* **325**, 79–85 (2000).

[20] Case, D. A. *et al.* Amber 2018 (2018). University of California, San Francisco.
