# Supplementary material for: Protein control of photochemistry and transient intermediates in phytochromes
Source: Nat Commun. 2022 Nov 11;13:6838. doi: 10.1038/s41467-022-34640-8 (PMC9652276; doi:10.1038/s41467-022-34640-8)
Supplement: Supplementary file 3 — Description of additional Supplementary File [file 41467_2022_34640_MOESM3_ESM.pdf]

Descriptions of additional Supplementary Files

Supplementary Movie 1: Each dot represents a Surface Hopping trajectory in the conformational space defined by the dihedrals D5 and D6. In this video, only some representative trajectories are represented. The left panel represents the first electronic excited state of the biliverdin chromophore, while the right panel the ground state. Blue dots are the reactive trajectories, while orange dots are the non-reactive trajectories.
