# Supplementary material for: Protein control of photochemistry and transient intermediates in phytochromes
Source: Nat Commun. 2022 Nov 11;13:6838. doi: 10.1038/s41467-022-34640-8 (PMC9652276; doi:10.1038/s41467-022-34640-8)
Supplement: Supplementary file 5 — Reporting Summary [file 41467_2022_34640_MOESM5_ESM.pdf]

nature portfolio

Corresponding author(s): GIACOMO SALVADORI
BENEDETTA MENNUCCI

Last updated by author(s): Oct 26, 2022

Reporting Summary

Nature Portfolio wishes to improve the reproducibility of the work that we publish. This form provides structure for consistency and transparency in reporting. For further information on Nature Portfolio policies, see our Editorial Policies and the Editorial Policy Checklist.

Statistics

For all statistical analyses, confirm that the following items are present in the figure legend, table legend, main text, or Methods section.

n/a	Confirmed	
☐	☒	The exact sample size (*n*) for each experimental group/condition, given as a discrete number and unit of measurement
☒	☐	A statement on whether measurements were taken from distinct samples or whether the same sample was measured repeatedly
☒	☐	The statistical test(s) used AND whether they are one- or two-sided<br>*Only common tests should be described solely by name; describe more complex techniques in the Methods section.*
☒	☐	A description of all covariates tested
☒	☐	A description of any assumptions or corrections, such as tests of normality and adjustment for multiple comparisons
☐	☒	A full description of the statistical parameters including central tendency (e.g. means) or other basic estimates (e.g. regression coefficient) AND variation (e.g. standard deviation) or associated estimates of uncertainty (e.g. confidence intervals)
☒	☐	For null hypothesis testing, the test statistic (e.g. *F*, *t*, *r*) with confidence intervals, effect sizes, degrees of freedom and *P* value noted<br>*Give P values as exact values whenever suitable.*
☒	☐	For Bayesian analysis, information on the choice of priors and Markov chain Monte Carlo settings
☒	☐	For hierarchical and complex designs, identification of the appropriate level for tests and full reporting of outcomes
☒	☐	Estimates of effect sizes (e.g. Cohen's *d*, Pearson's *r*), indicating how they were calculated

Our web collection on statistics for biologists contains articles on many of the points above.

Software and code

Policy information about availability of computer code

Data collection	Molecular Dynamics software: AMBER 2018<br>Surface Hopping and semiempirical method software: modified version of MOPAC2002 interfaced with TINKER 8.5<br>Other QM and QM/MM calculations: Gaussian 16 commercial software
Data analysis	CPPTRAJ V18.01<br>Python 3.7.13 with packages:<br>- seaborn 0.11.2<br>- scikit-learn 1.0.2<br>- scipy 1.7.3<br>- matplotlib 3.5.1

For manuscripts utilizing custom algorithms or software that are central to the research but not yet described in published literature, software must be made available to editors and reviewers. We strongly encourage code deposition in a community repository (e.g. GitHub). See the Nature Portfolio guidelines for submitting code & software for further information.

Data

Policy information about availability of data

All manuscripts must include a data availability statement. This statement should provide the following information, where applicable:

- Accession codes, unique identifiers, or web links for publicly available datasets
- A description of any restrictions on data availability
- For clinical datasets or third party data, please ensure that the statement adheres to our policy

The data supporting all the findings of this study are available within the article and its supplementary files. The source data generated in this study have been deposited in the Zenodo repository (\url{https://zenodo.org/record/7254721}). The PDB structures analyzed in this study can be accessed at https://www.rcsb.org/structure/4Q0J and https://www.rcsb.org/structure/5C5K

Human research participants

Policy information about studies involving human research participants and Sex and Gender in Research.

Reporting on sex and gender	Not applicable
Population characteristics	Not applicable
Recruitment	Not applicable
Ethics oversight	Not applicable

Note that full information on the approval of the study protocol must also be provided in the manuscript.

Field-specific reporting

Please select the one below that is the best fit for your research. If you are not sure, read the appropriate sections before making your selection.

☒ Life sciences ☐ Behavioural & social sciences ☐ Ecological, evolutionary & environmental sciences

For a reference copy of the document with all sections, see nature.com/documents/nr-reporting-summary-flat.pdf

Life sciences study design

All studies must disclose on these points even when the disclosure is negative.

Sample size	The number of QM/MM and MM molecular dynamics simulations were decided based on the available computational resources and sample representativity of the intermediate.<br>Sample size for QM/MM calculations (as it regards the IR spectroscopy simulation) were decided based on the available computational resources and sample representativity of the clusters.
Data exclusions	For the ground state simulations (QM/MM and MM MDs), only the "production" part of the trajectory is reported, i.e., minimization, heating, and equilibration are excluded from the analysis.
Replication	Overall, we have performed thousands of nonadiabatic trajectories to verify the photochemical mechanism and its robustness under different conditions (see text).<br>Four independent 5 ns-long QM/MM ground-state simulations were run to ensure reproducibility.<br>Ten independent 1 microsecond-long MM ground-state simulations were run to ensure reproducibility.
Randomization	No randomization was performed in our analysis.
Blinding	No blinding was performed, as blinding is not possible or applicable for molecular dynamics simulations

Reporting for specific materials, systems and methods

We require information from authors about some types of materials, experimental systems and methods used in many studies. Here, indicate whether each material, system or method listed is relevant to your study. If you are not sure if a list item applies to your research, read the appropriate section before selecting a response.

Materials & experimental systems

n/a	Involved in the study
☒	☐ Antibodies
☒	☐ Eukaryotic cell lines
☒	☐ Palaeontology and archaeology
☒	☐ Animals and other organisms
☒	☐ Clinical data
☒	☐ Dual use research of concern

Methods

n/a	Involved in the study
☒	☐ ChIP-seq
☒	☐ Flow cytometry
☒	☐ MRI-based neuroimaging

March 2021
